# CCT7 predicts poor prognosis and correlates with immune infiltration in colonic adenocarcinoma

**DOI:** 10.1007/s10735-026-10715-4

**Published:** 2026-01-28

**Authors:** Wenxu Li, Qizhong Shi, Yonghui Mu, Chenglei Li, Wenchao Zhao, Na Han

**Affiliations:** 1 Department of Oncology, The Second Affiliated Hospital of Zhengzhou University, Zhengzhou, Henan People’s Republic of China; 2 Department of Cardiothoracic Surgery, The Third Affiliated Hospital of Xinxiang Medical University, Xinxiang, Henan People’s Republic of China; 3 General Practice School of Xinxiang Medical University, Xinxiang, Henan People’s Republic of China; 4 Department of Physiology and Neurobiology, School of Basic Medical Sciences, Zhengzhou University, Zhengzhou, Henan People’s Republic of China

**Keywords:** Colonic adenocarcinoma, CCT7, Prognosis, Immune infiltration, Drug sensitivity

## Abstract

CCT7, a member of the t-complex polypeptide 1 chaperone family, facilitates ATP-dependent protein folding; however, its role in development and progression of malignant tumors remains unclear. This study aimed to characterize the expression pattern of CCT7 in colonic adenocarcinoma (COAD) and evaluate its role in the initiation and development of COAD. Public bioinformatic databases were analyzed to assess CCT7 expression in COAD, and these findings were validated using human clinical specimens through immunohistochemistry (IHC) assay. The prognostic significance of CCT7 was examined using Kaplan–Meier method and Cox regression analysis. Gene Ontology-Biological Process (GO-BP) and Kyoto Encyclopedia of Genes and Genomes (KEGG) enrichment analyses were performed to explore the potential biological functions and downstream pathways associated with CCT7. CCK-8, colony formation and Transwell assays were conducted to determine the impact of CCT7 on cell proliferation, migration and invasion in COAD cell lines. Associations between CCT7 expression and immune cell infiltration or drug sensitivity were evaluated using single-sample gene set enrichment analysis and correlation analysis. Finally, immune checkpoint inhibitor therapy scores and their relationship with CCT7 expression were assessed using data from The Cancer Immunome Atlas. CCT7 expression was significantly up-regulated statistically in COAD tissues compared with normal colonic tissues (*P*< 0.05) and elevated CCT7 levels were associated with poorer prognosis of COAD patients (*P*< 0.05). GO-BP enrichment analysis indicated that CCT7 was primarily involved in the processes related to cell proliferation and microtubule organization (*P*< 0.05). Consistently, functional assays confirmed that CCT7 knockdown inhibited COAD cell proliferation, migration, and invasion (*P*< 0.05). CCT7 expression showed a negative correlation with infiltration of most immune cell types (*P*< 0.05) and demonstrated no significant association with predicted responses to PD-1 and CTLA-4 inhibitor therapies (*P*> 0.05). Moreover, drug sensitivity analyses showed that CCT7 affected the sensitivity of COAD samples to several anti-cancer drugs (*P*< 0.001). KEGG enrichment analysis revealed that CCT7 was associated with multiple pathways (*P*< 0.05). CCT7 may function as an oncogenic driver that promotes the malignant phenotype of COAD and represents a promising prognostic biomarker. It may also provide a valuable reference for guiding clinical therapeutic strategies in COAD.

## Introduction

Colorectal cancer (CRC) is the third most common gastrointestinal malignancy worldwide (Morgan et al. 2023). According to the recent estimates from the International Agency for Research on Cancer, there were approximately 20 million new cancer cases and 9.7 million cancer-related deaths worldwide in 2022, with CRC accounting for 9.6% of new cancer cases and 9.3% of cancer-related deaths. Moreover, approximately 2 million new cases and an estimated 900,000 fatalities were reported (Bray et al. 2024). Given this significant disease burden, research on the molecular mechanisms for the development and progression of CRC is advancing in multiple dimensions. Metabolic reprogramming influences tumor progression by modulating the immune microenvironment (Chen et al. 2024b). Specifically, mitochondrial metabolic reprogramming, by disrupting oxidative phosphorylation, fatty acid oxidation, and redox homeostasis, has emerged as a key strategy for overcoming drug resistance (Qiu et al. 2025). Additionally, the mitochondrial-endoplasmic reticulum contact site facilitates calcium ion transport and lipid synthesis, thereby driving tumor cell proliferation (Xiao et al. 2026). Key enzymes in fatty acid metabolism also promote metastasis by regulating reactive oxygen species, matrix metalloproteinases (MMPs), and epithelial-mesenchymal transition (EMT) (Li et al. 2024). EMT, central to invasion and colonization, endows cancer cells with stem cell-like properties and enables immune evasion (Nie et al. 2025). Elucidating these mechanisms provides a vital theoretical foundation for developing CRC therapies. Although remarkable progress has been made in elucidating the onset and progression of CRC, the precise molecular and cellular mechanisms driving its development are yet to be fully understood.

Colonic adenocarcinoma (COAD) represents the predominant pathological subtype of CRC (Yang et al. 2022b). Despite substantial advances in treatment strategies, the overall survival (OS) of patients with COAD remains unsatisfactory. Therefore, elucidating the pathogenic mechanisms of COAD and identifying reliable prognostic biomarkers and therapeutic targets remain urgent clinical priorities.

Emerging evidence suggests that molecular chaperones play critical roles in tumorigenesis and progression, among which the chaperonin containing t-complex 1 (CCT) has attracted increasing attention due to its involvement in regulating key oncogenic processes (Xu et al. 2024). CCT is an essential cytoplasmic molecular chaperone in eukaryotic cells composed of eight distinct subunits (CCT1-CCT8) (Brackley and Grantham 2009; Vallin and Grantham 2019). Actin and tubulin, the main folding substrates of CCT, require the CCT complex for proper folding both in vivo and in vitro (Valpuesta et al. 2002). Given that cytoskeletal remodeling, metabolic reprogramming, and proteostasis are central to oncogenesis, there is increasing interest in understanding the contributions of CCT to tumor development (Parker et al. 2014; Khromova et al. 2024). Previous studies have revealed that CCT3 promotes lung adenocarcinoma (LUAD) via glycolysis and EIF3G-mediated translation (Chen et al. 2022). CCT4 enhances glucose metabolism and chemoresistance in esophageal squamous cell carcinoma through the AMPK/AKT/NRF2 pathway (Fang et al. 2022), and tumor-promoting roles of CCT1, CCT2, CCT5, CCT6A, and CCT8 have also been reported (Yang et al. 2018; Chang et al. 2020; Tang et al. 2020; Cai et al. 2022; Li et al. 2022). However, the function of CCT7, particularly in COAD, remains poorly understood. Although bioinformatics analyses suggest that CCT7 mRNA levels are elevated in CRC tissues compared with adjacent normal tissues (Lim et al. 2022), its biological role and regulatory mechanisms in COAD have not yet been elucidated.

Tumor development is closely associated with the tumor microenvironment (TME), in which immune infiltrating cells play a critical regulatory role (Guo and Xu 2024; Erasha et al. 2025). Immune checkpoints, molecules expressed on immune cells, play an important role in regulating immune cell activity and affecting tumor prognosis (Franzin et al. 2020; Arafat Hossain 2024). The emergence and significant efficacy of immunotherapy have further highlighted the value of immune-related biomarkers. However, the relationship between CCT7 and immune infiltration cells or immune checkpoint regulation in COAD remains largely unexplored. Therefore, this study aims to characterize CCT7 expression in COAD, investigate its biological roles, prognostic significance, association with immune infiltration, and potential implications for therapy in COAD.

## Materials and methods

### Patients and tissue samples

All clinical tissue specimens used in this study were sourced from the Second Affiliated Hospital of Zhengzhou University and verified by histopathology. We conducted immunohistochemical staining on 60 COAD tissues and their paired adjacent normal colon tissues. Written informed consent was obtained from the patients for the use of these clinical materials in research, and approval was granted by the Institutional Research Ethics Committee of the Second Affiliated Hospital of Zhengzhou University. The study adhered to all relevant ethical regulations regarding human participants. The clinical characteristics of COAD patients are shown in Table 1.

**Table 1:** Clinical characteristics of COAD patients from COAD clinical specimens

Characteristics	Number of samples (%)
*Age(years)*	
≤ 65	30 (50.0)
> 65	30 (50.0)
*Gender*	
Male	30 (50.0)
Female	30 (50.0)
*M stage*	
M0	54 (90.0)
M1	6 (10.0)
*N stage*	
N0	39 (65.0)
N1	15 (25.0)
N2	6 (10.0)
*T stage*	
T1	4 (6.7)
T2	9 (15.0)
T3	40 (66.6)
T4	7 (11.7)
*Tumor location*	
Right	30 (50.0)
Left	30 (50.0)
*Loss of MMR protein*	
No	43 (71.7)
Yes	17 (28.3)
*Lymphatic invasion*	
No	28 (46.7)
Yes	32 (53.3)
*Non nodal tumor deposits*	
No	43 (71.7)
Yes	17 (28.3)
*PostopTreat*	
No	14 (23.3)
Yes	46 (76.7)
*Venous invasion*	
No	43 (71.7)
Yes	17 (28.3)
*Grade*	
Well differentiated	11 (18.3)
Moderately differentiated	40 (66.7)
Poorly differentiated	9 (15.0)
*Overall survival*	
Alive	42 (70.0)
Dead	18 (30.0)

### Patient characteristics

Inclusion criteria consisted of patients with confirmed pathological diagnosis of COAD and complete clinico-pathological data. Exclusion criteria included samples lacking essential pathological information, patients with a history of other malignancies, and those who received prior radiotherapy or chemotherapy.

### TCGA and GEO data collection and processing

The RNA-sequencing data of COAD and the related clinical data (Table 2) were obtained from The Cancer Genome Atlas (TCGA) (https://portal.gdc.cancer.gov/repository) (Weinstein et al. 2013). TCGA-COAD was used for the investigation of CCT7 expression (log_2_(FPKM + 1)) and its prognostic value, Gene Ontology-Biological Process (GO-BP) enrichment analysis, Kyoto Encyclopedia of Genes and Genomes (KEGG) enrichment analysis, immune-related analysis, and drug sensitivity.

**Table 2:** Clinical characteristics of COAD patients from TCGA database

Characteristics	Number of samples (%)
*Age(years)*	
≤ 68	153 (45.5)
> 68	183 (54.5)
*Gender*	
Male	180 (53.6)
Female	156 (46.4)
*M stage*	
M0	280 (83.3)
M1	56 (16.7)
*N stage*	
N0	201 (59.8)
N1	76 (22.6)
N2	59 (17.6)
*T stage*	
T1	8 (2.4)
T2	60 (17.9)
T3	231 (68.8)
T4	37 (11.0)
*Tumor location*	
Right	140 (41.7)
Left	112 (33.3)
Unknown	84 (25)
*Loss of MMR protein*	
No	230 (68.5)
Yes	37 (11.0)
Unknown	69 (20.5)
*Lymphatic invasion*	
No	176 (52.4)
Yes	130 (38.7)
Unknown	30 (8.9)
*Non nodal tumor deposits*	
No	133 (39.6)
Yes	26 (7.7)
Unknown	177 (52.7)
*Cancer status*	
Tumor free	206 (61.3)
With tumor	55 (16.4)
Unknown	75 (22.3)
P*ostopTreat*	
No	184 (54.8)
Yes	106 (31.5)
Unknown	46 (13.7)
*Venous invasion*	
No	221 (65.8)
Yes	74 (22.0)
Unknown	41 (12.2)
*Overall survival*	
Alive	268 (79.8)
Dead	68 (20.2)

Microarray data of GSE39582, GSE41258, GSE37364, GSE44076, GSE41328 and GSE17538 were retrieved from Gene Expression Omnibus (GEO) (https://www.ncbi.nlm.nih.gov/geo/) (Lin et al. 2006; Sheffer et al. 2009; Smith et al. 2010; Marisa et al. 2013; Solé et al. 2014; Valcz et al. 2014). For the GSE39582 dataset, raw CEL files were preprocessed using the Robust Multi-array Average (RMA) algorithm, followed by batch effect correction with the ComBat algorithm from the sva package in R. For the GSE41258, GSE37364, GSE44076, GSE41328 and GSE17538 datasets, the raw CEL files were preprocessed using the RMA algorithm. GSE39582 was applied for CCT7 expression, GO-BP enrichment analysis and KEGG enrichment analysis. GSE39582, GSE41258, GSE37364, GSE44076 and GSE41328 were analyzed for CCT7 expression. GSE17538 was taken for survival analysis.

### Cell lines and culture

The human COAD cell lines (RKO, HCT-116) and the normal colon epithelial cell line (NCM460) were obtained from the Cell Bank of the Chinese Academy of Sciences (Shanghai, China). RKO and HCT-116 cells were cultured in 1640 medium, while NCM460 cells were cultured in DMEM medium. All media were supplemented with 10% fetal bovine serum (FBS) (Genial Biologicals, Inc.) and 1% penicillin–streptomycin mixture. Cells were incubated in a humidified atmosphere with 5% CO₂ at 37 °C.

### Construction of siRNA knockdown target gene cell lines

Transient transfection siRNA (siCCT7, siNC) was provided by GenePharma (GenePharma, Shanghai, China). Cultured COAD cells (RKO, HCT-116) were seeded in a six-well plate at equal densities using complete medium. When cell confluence reached approximately 50%, the mixed reagent was mixed with 100 μL of buffer per well, with 5.85 μL of transfection reagent (Polyplus-transfection®, jetPRIME®, France) added. The mixture was incubated with 4.5 μL of siRNA at room temperature for 10 min and then added to the six-well plate.

The sequences of small interfering RNAs (siRNAs) targeting CCT7 were as follows:

siRNA-1: sense 5′-CAUUCUCUAUGACAAGUUAGA-3′, antisense 5′-UAACUUGUCAUAGAGAAUGUU-3′.

siRNA-2: sense 5′-CCAUCAAGAAUGAUUCAGUGG-3′, antisense 5′-ACUGAAUCAUUCUUGAUGGCC-3′. The negative control siRNA sequences were as follows: sense 5′-UUCUCCGAACGUGUCACGUTT-3′, antisense 5′-ACGUGACACGUUCGGAGAATT-3′.

### Quantitative real-time polymerase chain reaction (qRT-PCR)

Total RNA was extracted using TRIzol reagent (Invitrogen, USA) following the manufacturer’s instructions. cDNA was synthesized using a reverse transcription kit (Vazyme, Nanjing). qRT-PCR was performed in a 20 μL reaction mixture using SYBR Green qPCR Master Mix (Vazyme, Nanjing) and the Applied ABI QuantStudio 3 Real-Time PCR System (Thermo Fisher Scientific, Inc.). The primer pairs used for qRT-PCR were: CCT7 forward primer 5′-GCTGGTGTTGCATTCAAGAAG-3′ and reverse primer 5′-TTGCCTGATAATCCTCAACTGTG-3′; GAPDH forward primer 5′-GGAGCGAGATCCCTCCAAAAT-3′ and reverse primer 5′-GGCTGTTGTCATACTTCTCATGG-3′. Relative expression was normalized to the internal reference gene GAPDH using the 2-ΔΔCt method.

### Western blotting

Cells were lysed using a radio-immunoprecipitation assay buffer (Cwbio, Jiangsu, China), and the protein concentration was determined using a bicinchoninic acid protein assay kit (Beyotime, Shanghai, China). Protein samples (30 μg/lane) were separated by sodium dodecyl sulfate–polyacrylamide gel electrophoresis on 10% gels and transferred onto polyvinylidene fluoride membranes. The membranes were blocked with 10% skim milk at room temperature for 2 h, then incubated overnight at 4 °C with primary antibodies against CCT7 (15,994-1-AP; Proteintech, China) and β-actin (AC026; Abclonal, China). After incubation, the membranes were treated with anti-rabbit horseradish peroxidase secondary antibody (SA00001-2; Proteintech, China) at room temperature for 1 h. Protein bands were visualized using an enhanced chemiluminescence reagent kit (Abbkine Scientific Co., Ltd, China) and scanned with an imaging system (ProteinSimple, Inc.). Protein band densities were quantified using ImageJ software v1.8.0.

### Cell counting kit-8 (CCK-8) assay

Cell proliferation ability was assessed using the CCK-8 assay (Abbkine Scientific Co., Ltd, China). Transfected siNC and siCCT7 cells were seeded in 96-well plates and incubated at 37 °C for 24, 48, 72, and 96 h. After adding 10 μL of CCK-8 reagent to each well and incubating in the dark for 1.5 h, absorbance was measured at 450 nm.

### Colony formation assays

Colony formation assay was employed to evaluate the cloning capability of COAD cells with stable CCT7 knockdown. The transfected siNC and siCCT7 cells were seeded in six-well plates at approximately 1000 cells per well. After 15 days of culture, RKO and HCT-116 cells were fixed with 4% fixative solution and stained with 0.1% crystal violet staining solution (Solarbio Life Sciences, Beijing, China). Colonies were washed with distilled water, photographed, and counted using ImageJ software.

### Cell invasion assay

RKO and HCT-116 cells were digested and resuspended in serum-free 1640 medium at a density of 1 × 10^5^ cells/mL. Cell invasion ability was assessed using the Transwell assay. The lower chamber of each well was filled with 600 μL of complete medium containing 10% FBS. Matrigel was diluted 1:8 with serum-free medium, and 100 μL of this mixture was added to the upper chamber. Subsequently, 200 μL of the cell suspension was seeded into the upper chamber of a 24-well Transwell insert with a polycarbonate membrane (8.0 μm pore size). After 48 h of incubation, cells on the lower surface of the membrane were fixed, stained, and counted.

### Cell migration assay

RKO and HCT-116 cells were digested and resuspended in serum-free 1640 medium at a density of 1 × 10^5^ cells/mL. Cell migration ability was assessed using the Transwell assay. The lower chamber of each well was filled with 600 μL of complete medium containing 10% FBS. Subsequently, 200 μL of the cell suspension was seeded into the upper chamber of a 24-well Transwell insert with a polycarbonate membrane (8.0 μm pore size). After 48 h of incubation, cells on the lower surface of the membrane were fixed, stained, and counted.

### Immunohistochemistry (IHC) assay

The 60 COAD specimens were cut into 4-μm sections and fixed on glass slides for microscopy. The tissue sections on the slides were then deparaffinized and rehydrated using gradient concentrations of xylene and ethanol. Next, the slides were immersed in boiling Tris/ ethylenediaminetetraacetic acid (pH 9.0) for 20 min for antigen retrieval. The slides were subsequently immersed in 3% H₂O₂ for 10 min to inhibit endogenous peroxidase. Then, the slides were incubated with a primary antibody against CCT7 (1:250; 15,994-1-AP; Proteintech, China), followed by the HRP secondary antibody (RGAR011; Proteintech, China), and were washed three times with phosphate-buffered saline. Finally, the sections were stained with 3,3′-diaminobenzidine and substrate chromogen (Dako) for 2 min at room temperature and then counterstained with hematoxylin for 40 s. Slides incubated only with the secondary antibody without the primary antibody were used as the negative control.

The staining of each section was evaluated by two independent researchers blinded to the clinicopathological parameters. Immunoreactivity for CCT7 expression was evaluated using semi-quantitative immunoreactivity score (IRS) system, according to the intensity of staining (IS) (0-negative; 1-weak; 2-moderate; 3-strong) and area of positivity (AP) (0, 0–25%; 1, 25–50%; 3, 50–75%; 4, 75–100%), and recorded with H-score. The final H-score was obtained by multiplying the IS and AP. In this study, CCT7 expression was regarded as low expression when the score was < 6, and high expression when the score was ≥ 6. The immunohistochemical staining was assessed by two separate pathologists who were blinded to patients’ information.

### GO-BP and KEGG enrichment analyses

Spearman’s test was conducted using R software (v4.1.2) based on TCGA-COAD and GSE39582. CCT7-related genes (*P* < 0.05 and absolute value of correlation coefficient ≥ 0.35) were subjected to GO-BP and KEGG enrichment analyses using the Metascape database (accessed on 15 May 2025) (http://metascape.org/).

### Immune infiltration analysis

Single-sample gene set enrichment analysis (ssGSEA) was applied for determination of immune infiltration based upon the immune gene sets (Charoentong et al. 2017). ssGSEA for immune infiltration was performed using R (v4.1.2). The ssGSEA was implemented with the following R packages: GSVA (v1.42.0), Biobase (v2.54.0), genefilter (v1.76.0), and stringr (v1.5.1).

### Evaluation of drug sensitivity

Drug sensitivity prediction for the TCGA-COAD dataset was performed using the oncoPredict (v1.2) in the R environment (v4.1.2). The analysis utilized ridge regression models trained on GDSC2 database data (GDSC2_Expr.rds and GDSC2_Res.rds) and applied them to the TCGA-COAD dataset. The resulting sensitivity scores represent predicted drug response, where higher scores indicate lower sensitivity. All 199 drugs were evaluated using default parameters.

### Calculation of the scores for immune checkpoint inhibitor (ICI) therapy

The immunophenoscore (IPS), a well-validated biomarker that predicts response to ICI therapy, was obtained from The Cancer Immunome Atlas (TCIA) (https://tcia.at/patients). For each sample in the TCGA-COAD dataset, four types of IPS were calculated, i.e., IPS-CTLA4-neg-PD1-neg, IPS-CTLA4-neg-PD1-pos, IPS-CTLA4-pos-PD1-neg, and IPS-CTLA4-pos-PD1-pos. These scores predict the potential efficacy of different treatment scenarios, i.e., the absence of both CTLA-4 and PD-1 blockade, PD-1 blockade alone, CTLA-4 blockade alone, and combined CTLA-4 and PD-1 blockade, respectively.

### Statistical analysis

Statistical analyses were performed using SPSS 26.0 (IBM, USA) and GraphPad Prism 8 (version 8.0.2, GraphPad Software, San Diego, CA, USA). Continuous variables are presented as mean ± standard deviation (SD). Comparison between two groups was conducted using the independent-samples t-test or paired-samples t-test, whereas one-way analysis of variance (ANOVA) was applied for comparison among three or more groups. Non-parametric tests were used when data did not meet the assumptions of normality or homogeneity of variance. The Kaplan–Meier method with log-rank test was employed to evaluate the impact of CCT7 on COAD prognosis. Cox regression analysis was performed for univariate and multivariate analyses of CCT7 and clinicopathological parameters. In univariate regression, variables with a p-value less than 0.2 were included in the multivariate regression. Pearson correlation was used for normally distributed samples, while Spearman’s correlation was applied for non-normally distributed ones. Categorical variables are presented as frequencies and percentages (%), and inter-group comparisons were performed using the χ^2^ test. *P* < 0.05 was considered statistically significant.

## Results

### Abnormal high expression of CCT7 in COAD

**Fig. 1:**
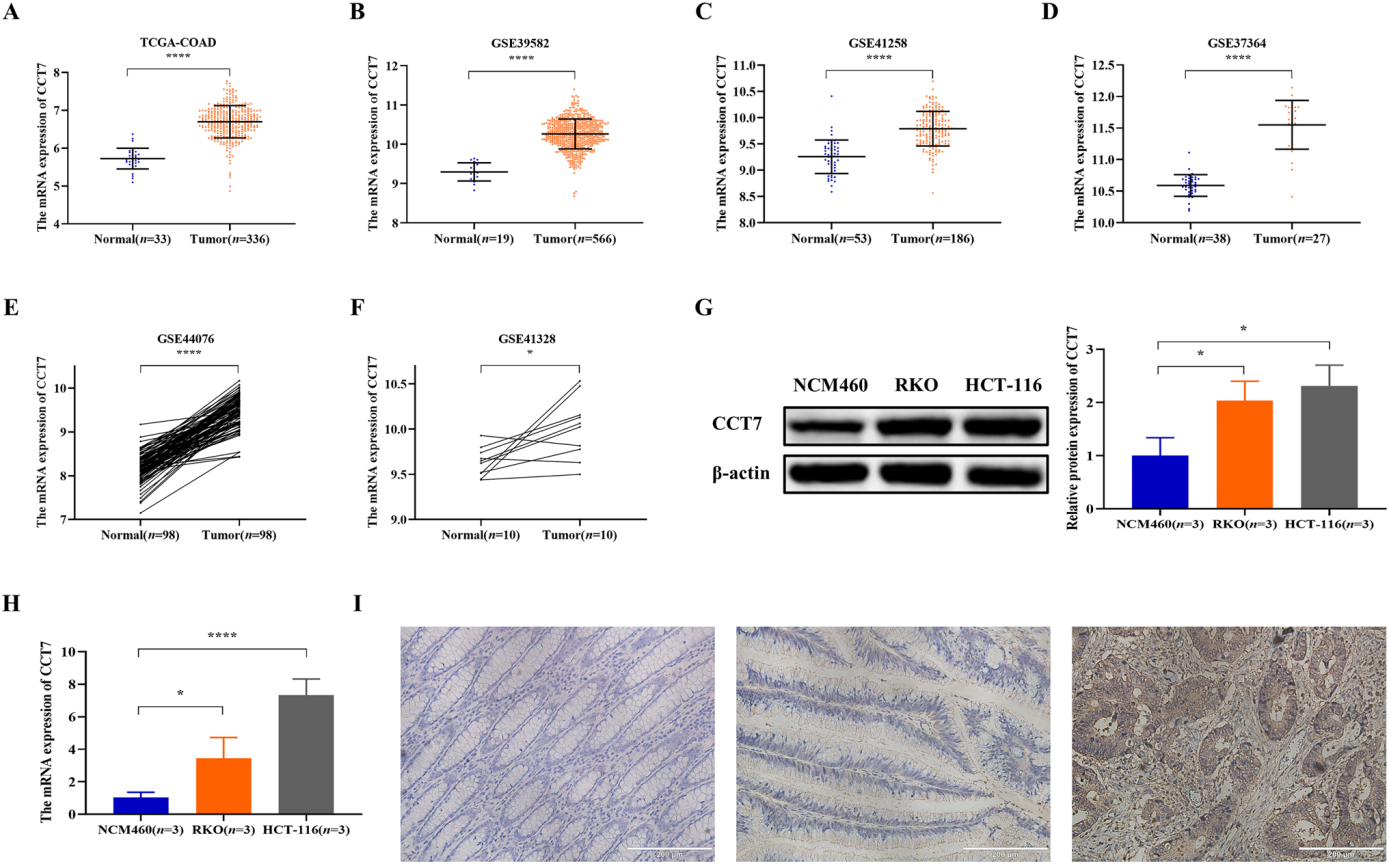
CCT7 was upregulated in human COAD tissue. **A–F** Analyses based on TCGA-COAD (**A)**, GSE39582 (**B)**, GSE41258 (**C)**, GSE37364 (**D)**, GSE44076 (**E)** and GSE41328 (**F)** datasets demonstrated significantly higher CCT7 mRNA expression in COAD cancer tissues than in paracancerous tissues. **G** and **H** Increased protein (**G)** and mRNA (**H)** levels of CCT7 in the COAD cell lines RKO and HCT-116 compared with normal colon cell line NCM460. **I** Representative images of IHC detection for CCT7 expression in cancerous and paired noncancerous samples obtained from COAD patients. The left image, showing normal colon tissues, indicated that CCT7 was not detected, and the middle and right images demonstrated the low and high expression of CCT7 respectively in COAD tissues. All photomicrographs were captured at × 200 magnification. The data presented are representative of at least three independent experiments, with values expressed as the mean ± SD. Independent-samples t-test, paired-samples t-test and non-parametric tests were used for two-group comparisons, and one-way ANOVA and non-parametric tests were applied for multi-group comparisons. ****P < 0.0001, *P < 0.05

The expression of CCT7 in COAD was assessed using TCGA-COAD and GEO datasets. The analyses revealed significantly higher CCT7 mRNA levels in COAD cancer tissues than in pericancerous tissues (Fig. 1A–F, *P*<0.05). To validate these findings, western blotting and qRT-PCR were performed to measure CCT7 expression in COAD cell lines. Consistent with the bioinformatics results, both CCT7 protein and mRNA levels were markedly elevated in COAD cells (RKO and HCT-116 cells) compared with normal colon epithelial cells (NCM460 cells) (Fig. 1G and H, *P*< 0.05). Representative immunohistochemical images of tumor tissues and corresponding adjacent normal tissues are shown in Fig. 1I. H-score evaluation demonstrated that CCT7 protein was highly expressed in 38 of 60 (63.3%) COAD samples compared with 11 of 60 (18.3%) adjacent normal samples, with a statistically significant difference (*χ*^*2*^ = 25.145, *P* < 0.0001).

### High expression of CCT7 in COAD tissues is significantly associated with poor prognosis

**Fig. 2:**
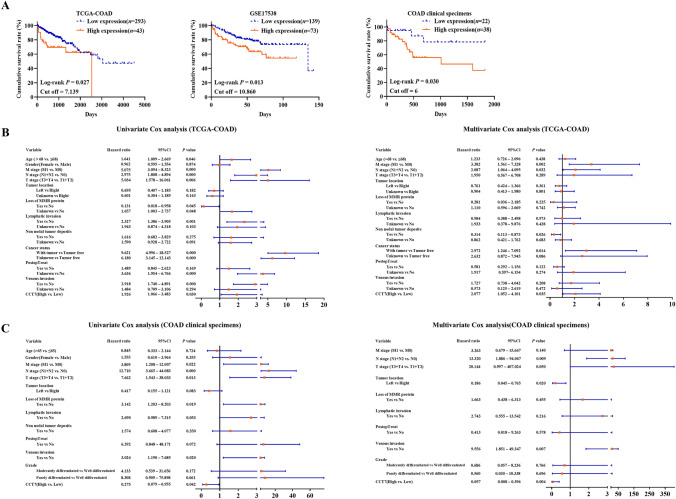
CCT7 overexpression in COAD tissues predicted poor prognosis. **A** Kaplan–Meier and log-rank were used to analyze the correlation between the expression of CCT7 and the prognosis of COAD patients. **B** and **C** Univariate and multivariate Cox regression analyses based upon TCGA-COAD dataset (**B)** and COAD clinical specimens (**C)**. (MMR: Mismatch Repair Proteins; PostopTreat: Postoperative treatment)

Kaplan–Meier and log-rank analyses based on TCGA-COAD and GSE17538 datasets, as well as COAD clinical specimens, revealed that patients with high CCT7 expression had significantly shorter survival time compared with those with low CCT7 expression (Fig. 2A, *P*< 0.05). To evaluate whether CCT7 expression serves as an independent prognostic factor, Cox regression analyses were performed using TCGA-COAD dataset and COAD clinical specimens. In TCGA-COAD dataset, univariate analysis identified age, M stage, N stage, T stage, loss of MMR protein, lymphatic invasion, cancer status, PostopTreat, venous invasion, and CCT7 expression as predictors of prognosis. Multivariate analysis indicated that M stage, N stage, non-nodal tumor deposits, cancer status, and CCT7 expression were independent predictors of prognosis (Fig. 2B, *P*< 0.05). By using COAD clinical specimens, univariate analysis showed that M stage, N stage, T stage, loss of MMR protein, venous invasion, and CCT7 expression were predictors of prognosis. Multivariate analysis identified that N stage, tumor location, venous invasion, and CCT7 expression were independent predictors of prognosis (Fig. 2C, *P*< 0.05).

### GO-BP enrichment analysis for the biological functions of CCT7

**Fig. 3:**
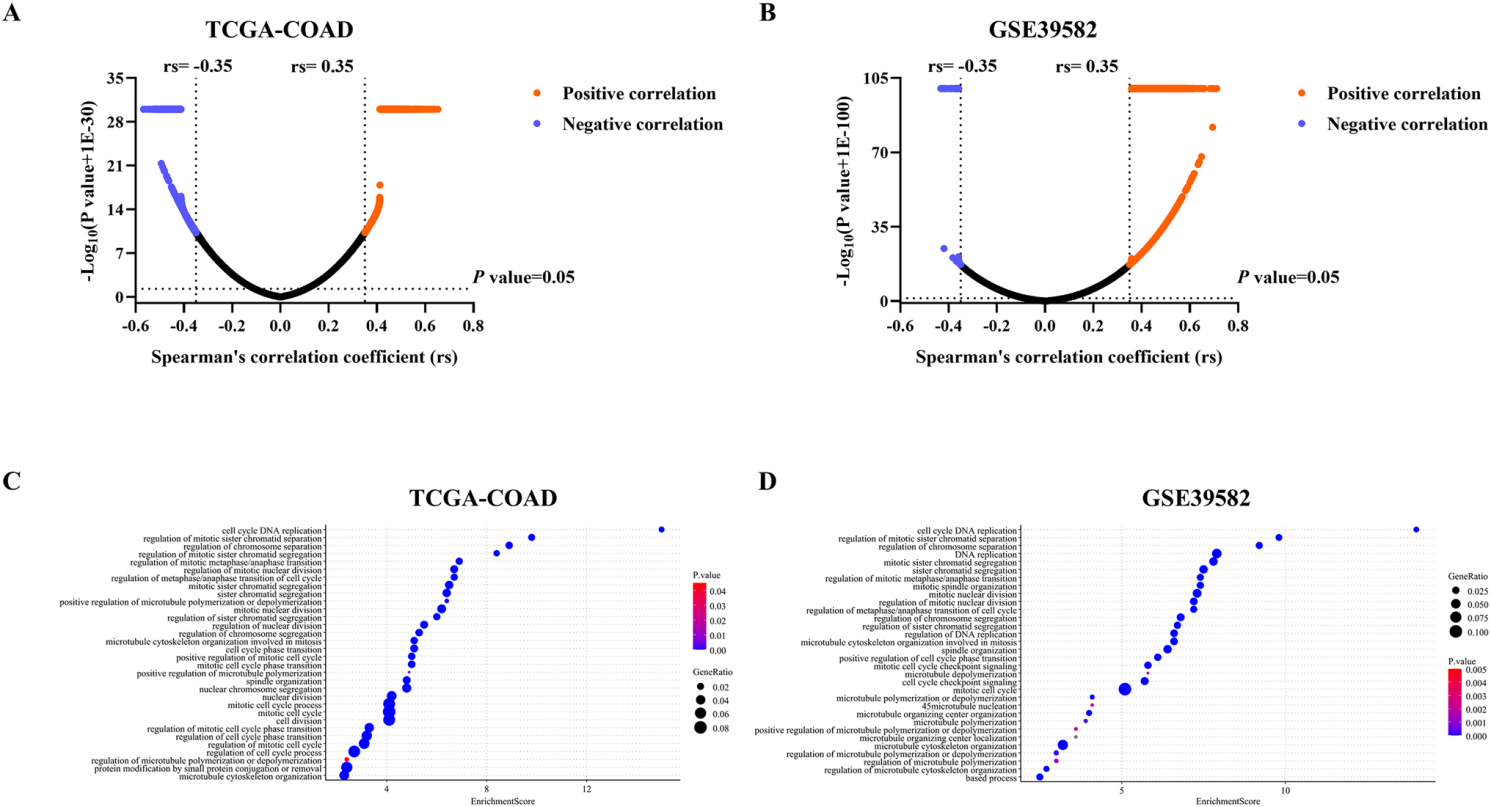
GO-BP enrichment analysis of genes associated with CCT7. **A** and **B** The correlation between each gene and CCT7 expression was analyzed using the TCGA-COAD (**A)** and GSE39582 (**B)** datasets. **C** and **D** GO-BP enrichment analysis of CCT7 was conducted using the TCGA-COAD (**C)** and GSE39582 (**D)** datasets. Spearman was used for correlation. (*rs*: correlation coefficient)

To explore potential functions of CCT7 in COAD development, GO-BP enrichment analysis was performed using the TCGA-COAD and GSE39582 datasets. First, the correlations between CCT7 expression and other genes in the two datasets were analyzed. Genes with |*rs*|≥ 0.35 and *P*< 0.05 were selected for enrichment analysis (Fig. 3A and B). The results indicated that CCT7 may be involved in regulating biological processes related to cell proliferation and microtubule activities in COAD (Fig. 3C and D,  *P*< 0.05). These findings suggest that CCT7 may play important roles in regulating COAD cell proliferation and metastasis.

### Knocking down CCT7 inhibited the proliferation, migration, and invasion of COAD cells

**Fig. 4:**
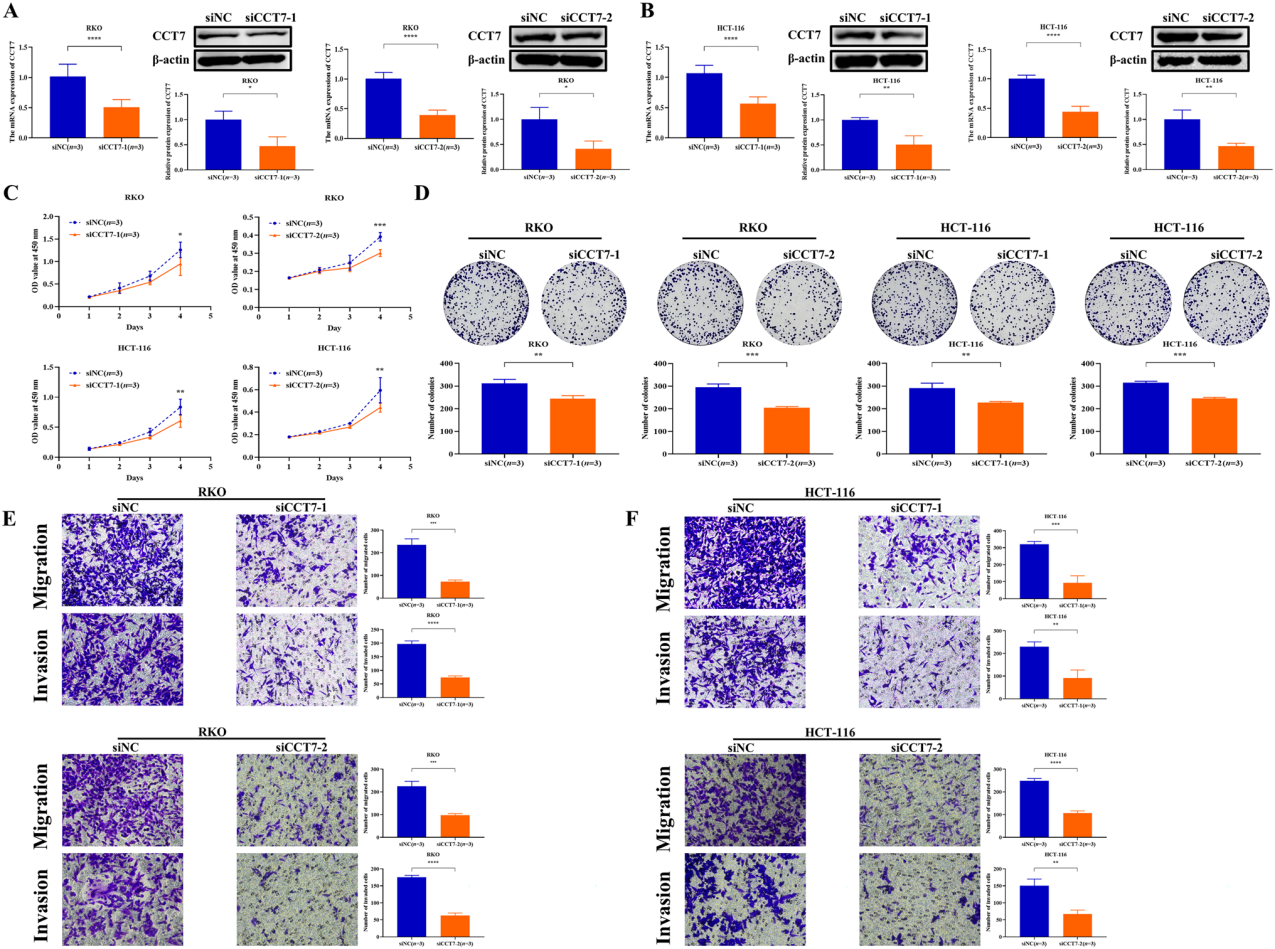
Downregulation of CCT7 repressed the proliferation, migration and invasion of COAD cells. **A** and **B** mRNA and protein expression levels of CCT7 in the RKO (**A)** and HCT-116 (**B)** cell lines. **C** The CCK-8 assay was performed to investigate the proliferation of COAD cells that were transfected with siNC or siCCT7. **D** Effect of siCCT7 on colony formation of COAD cells. **E** and **F** The migration and invasion abilities of RKO (**E**) and HCT-116 cells (**F)** were evaluated using Transwell analysis. All photomicrographs were captured at × 200 magnification. The data presented are representative of at least three independent experiments, with values expressed as the mean ± SD. Independent-samples t-test and non-parametric tests were used for two-group comparisons, and two-way ANOVA was applied for multi-group comparisons. ****P < 0.0001, ***P < 0.001, **P < 0.01, *P < 0.05

RKO and HCT-116 cells, which exhibit relatively high CCT7 expression, were selected for further study. After transfection with siCCT7 and a negative control siNC, the mRNA and protein levels of CCT7 were measured. The results showed that both mRNA and protein expression of CCT7 were markedly reduced in siCCT7-transfected cells compared with siNC-transfected controls (Fig. 4A and B, *P*< 0.05), indicating successful transfection of RKO and HCT-116 cells. CCT7 knockdown RKO and HCT-116 cells showed a significant reduction in proliferation compared with control cells at 96 h after transfection (Fig. 4C, *P*< 0.05). A colony formation assay was then used to evaluate long-term proliferative potential. Compared with the control group, siCCT7 transfected cells exhibited a marked decrease in the number of colonies, indicating impaired colony forming ability (Fig. 4D, *P*< 0.01). Transwell assays were performed to evaluate the migration and invasion of RKO and HCT-116 cells. Compared with the siNC group, the number of migrated and invaded cells was significantly reduced in the siCCT7 group (Fig. 4E and F, *P*< 0.01).

### Correlations between CCT7 expression and infiltrating immune cells

Based on the TCGA-COAD dataset, the associations of immune infiltration with CCT7 expression were investigated using ssGSEA algorithm and Spearman’s correlation analysis. The results showed that CCT7 expression was negatively associated with most immune cells, including central memory CD4 and CD8 T cells, effector memory CD4 and CD8 T cells, T follicular helper cell, type 1 and type 2 T helper cells (Table 3, *P*< 0.05). To validate the above findings, the correlations between CCT7 expression and immune cell marker genes were examined. CCT7 expression was negatively correlated with the expression of most immune cell markers (Table 4, *P*< 0.05). These results suggest that CCT7 expression levels may influence the immune infiltration characteristics of COAD.

**Table 3:** The correlation between CCT7 expression and immune infiltration in COAD

Immune cell	Correlation	*P* value
Activated B cell	− 0.182	0.001
Activated CD4 T cell	− 0.075	0.17
Activated CD8 T cell	− 0.038	0.483
Activated dendritic cell	− 0.119	0.029
CD56bright natural killer cell	− 0.04	0.47
CD56dim natural killer cell	0.06	0.269
Central memory CD4 T cell	− 0.302	< 0.001
Central memory CD8 T cell	− 0.174	0.001
Effector memeory CD4 T cell	− 0.199	< 0.001
Effector memeory CD8 T cell	− 0.241	< 0.001
Eosinophil	− 0.265	< 0.001
Gamma delta T cell	− 0.06	0.275
Immature B cell	− 0.238	< 0.001
Immature dendritic cell	− 0.116	0.034
Macrophage	− 0.123	0.024
Mast cell	− 0.108	0.048
MDSC	− 0.153	0.005
Memory B cell	− 0.217	< 0.001
Monocyte	− 0.086	0.117
Natural killer cell	− 0.238	< 0.001
Natural killer T cell	− 0.09	0.099
Neutrophil	− 0.013	0.807
Plasmacytoid dendritic cell	− 0.166	0.002
Regulatory T cell	− 0.128	0.019
T follicular helper cell	− 0.13	0.017
Type 1 T helper cell	− 0.149	0.006
Type 17 T helper cell	0.027	0.626
Type 2 T helper cell	− 0.182	0.001

**Table 4:** The correlation between CCT7 expression level and immune cell marker expression level in COAD

Immune cell	Gene	Correlation	*P* value
CD8^+^ T cell	CD8A	− 0.119	0.03
	CD8B	0.071	0.193
	PTPRC	− 0.355	< 0.001
T cell (general)	CD3D	− 0.02	0.721
	CD3E	− 0.192	< 0.001
	CD2	− 0.231	< 0.001
B cell	CD19	− 0.214	< 0.001
	CD79A	− 0.208	< 0.001
	CD27	− 0.202	< 0.001
	KRT20	− 0.054	0.325
Monocyte	CCL2	− 0.148	0.007
	CD14	− 0.073	0.183
	CD68	− 0.307	< 0.001
	IL10	− 0.116	0.033
	CSF1R	− 0.217	< 0.001
	CD86	− 0.215	< 0.001
TAM	CCL2	− 0.148	0.007
	CD68	− 0.307	< 0.001
	IL10	− 0.116	0.033
M1 Macrophage	NOS2	− 0.01	0.855
	CD80	− 0.198	< 0.001
	IRF5	− 0.082	0.132
	IL6	− 0.017	0.76
	FCGR1A	− 0.084	0.125
	PTGS2	− 0.231	< 0.001
M2 Macrophage	CD163	− 0.172	0.002
	MRC1	− 0.202	< 0.001
	VSIG4	− 0.076	0.163
	MS4A4A	− 0.12	0.028
Neutrophil	CEACAM8	0.072	0.19
	ITGAM	− 0.244	< 0.001
	FUT4	0.013	0.81
	CCR7	− 0.254	< 0.001
Natural killer cell	KIR2DS4	− 0.096	0.079
	NCAM1	− 0.358	< 0.001
	NCR1	− 0.224	< 0.001
Dendritic cell	CD1C	− 0.269	< 0.001
	NRP1	− 0.356	< 0.001
	THBD	− 0.29	< 0.001
	IL3RA	− 0.263	< 0.001
	ITGAX	− 0.266	< 0.001
Th1	TBX21	− 0.223	< 0.001
	IFNG	− 0.097	0.076
	STAT4	− 0.378	< 0.001
	STAT1	− 0.148	0.007
Th2	GATA3	− 0.218	< 0.001
	IL17A	0.041	0.456
	STAT6	− 0.096	0.078
Tfh	BCL6	− 0.324	< 0.001
Th17	STAT3	− 0.314	< 0.001
	qIL17A	0.041	0.456
Treg	FOXP3	− 0.246	< 0.001
	IL2RA	− 0.199	< 0.001
	CCR8	− 0.306	< 0.001
	STAT5B	− 0.2	< 0.001
	TGFB1	− 0.081	0.139
T cell exhaustion	PD-1	− 0.073	0.181
	CTLA-4	− 0.23	< 0.001
	LAG3	− 0.079	0.15
	HAVCR2	− 0.164	0.003
	GZMB	0.186	0.001

### Impact of CCT7 expression on the efficacy of ICI therapy

**Fig. 5:**
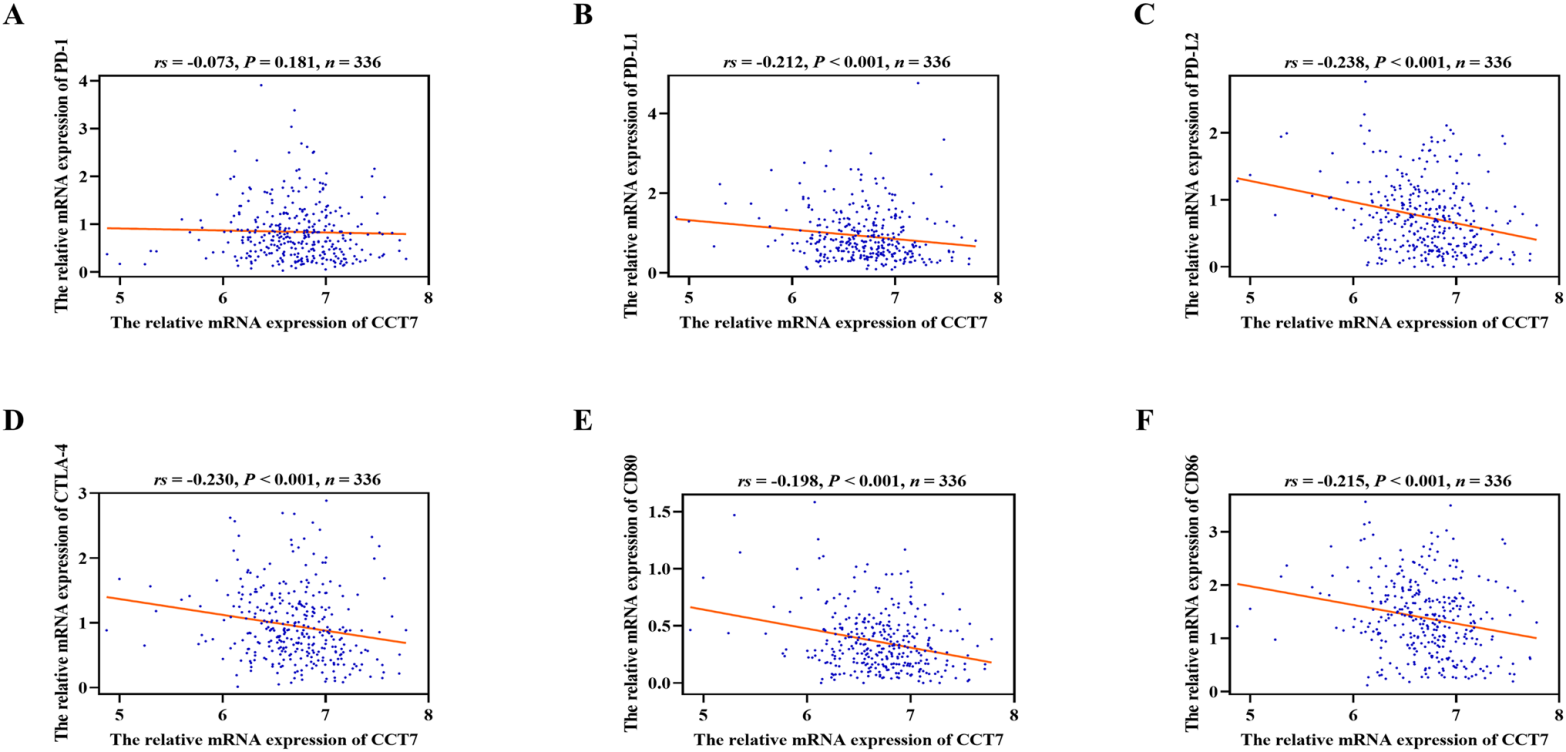
Impact of CCT7 expression on the efficacy of ICI therapy. **A–F** The correlation between PD-1 (**A**) and CTLA-4 (**D**), as well as their ligands PD-L1 (**B**), PD-L2 (**C**), CD80 (**E**) and CD86 (**F**) and CCT7 mRNA expression. Spearman was used for correlation

To investigate the impact of CCT7 expression on the therapeutic efficacy of ICI in COAD, correlations between CCT7 expression and the expression of immune checkpoints programmed cell death 1 (PD-1) and cytotoxic T-lymphocyte associated protein 4 (CTLA-4), and their ligands PD ligand 1 (PD-L1), PD ligand 2 (PD-L2), CD80 and CD86 were analyzed using the TCGA-COAD dataset. Although CCT7 expression showed no correlation with PD-1 (Fig. 5A, *P*= 0.181), it was negatively correlated with the expression of CTLA-4, PD-L1, PD-L2, CD80, and CD86 (Fig. 5B–F, *P*< 0.001). Subsequently, ICI therapy scores and their correlations with CCT7 expression were evaluated using the TCIA. The results showed that the CCT7 expression was not associated with the predicted responses of PD-1 and CTLA-4 inhibitor therapies (Table 5).

**Table 5:** The associations between CCT7 expression and IPS scores

Type of IPS	*rs*	*P* value
ips_ctla4_neg_pd1_neg	0.024	0.664
ips_ctla4_neg_pd1_pos	0.008	0.884
ips_ctla4_pos_pd1_neg	0.013	0.817
ips_ctla4_pos_pd1_pos	− 0.040	0.462

### Drug sensitivity analysis

**Fig. 6:**
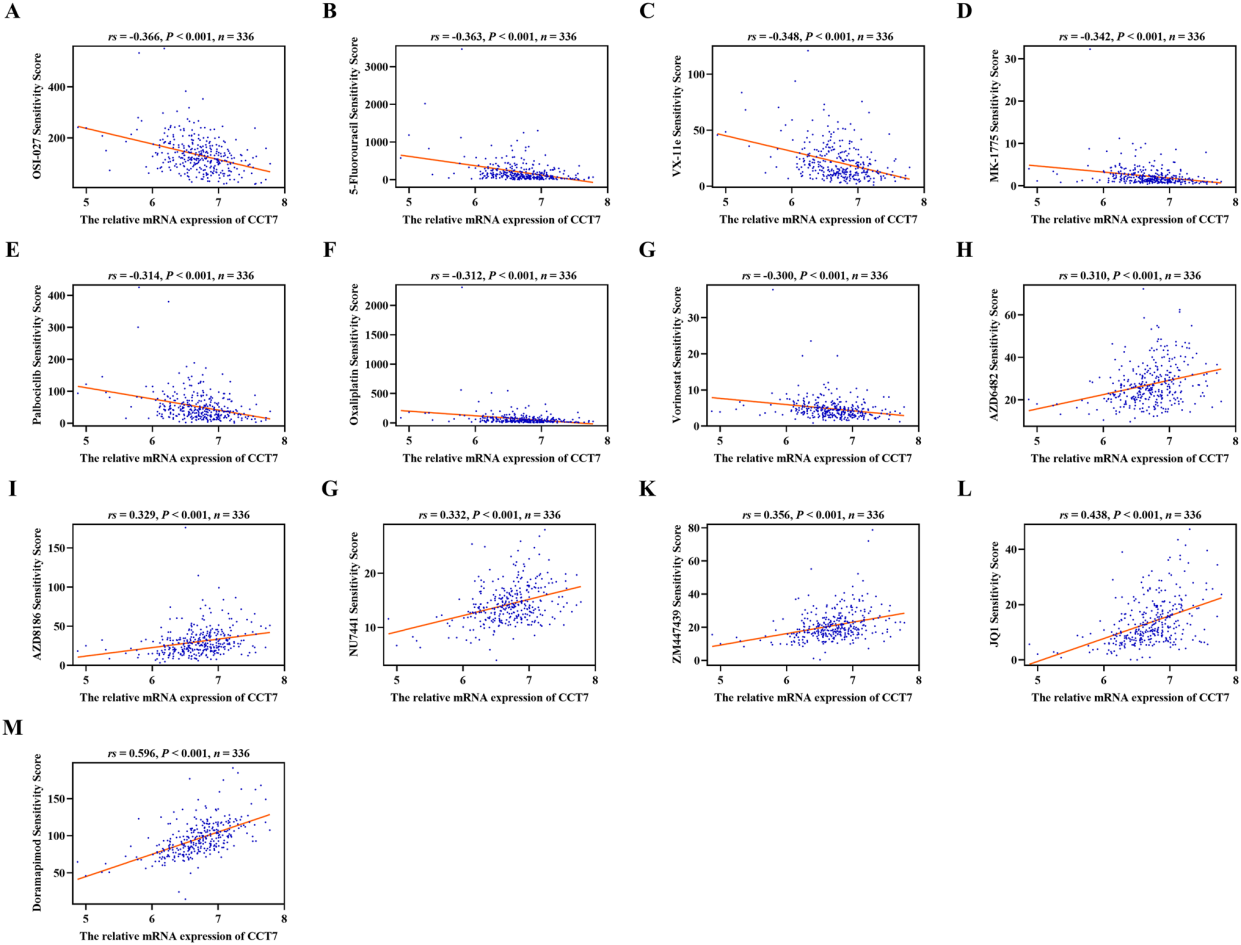
Associations between CCT7 expression and drug sensitivity based on TCGA-COAD dataset. **A–G** Negative correlations between the sensitivity scores of OSI-027 (**A**), 5-Fluorouracil (**B**), VX-11e (**C**), MK-1775 (**D**), Palbociclib (**E**), Oxaliplatin (**F**), and Vorinostat (**G**) and CCT7 mRNA expression. **H–M** Positive correlations between the sensitivity scores of AZD6482 (**H**), AZD8186 (**I**), NU7441 (**J**), ZM447439 (**K**), JQ1 (**L**), and Doramapimod (**M**) and CCT7 mRNA expression. Spearman was used for correlation

To investigate the impact of CCT7 expression on the efficacy of various anticancer drugs in COAD, the sensitivity scores of individual TCGA-COAD samples to multiple drugs were analyzed. In this scoring system, higher scores indicate lower drug sensitivity (i.e., reduced responsiveness). The correlations between drug sensitivity and CCT7 expression were then examined. The results showed that the CCT7 expression was negatively correlated with the sensitivity scores of OSI-027, 5-Fluorouracil, VX-11e, MK-1775, Palbociclib, Oxaliplatin, and Vorinostat (Fig. 6A–G, *P*< 0.001). Additionally, CCT7 expression was positively correlated with sensitivity scores of AZD6482, AZD8186, NU7441, ZM447439, JQ1, and Doramapimod (Fig. 6H–M, *P*< 0.001).

### High CCT7 expression correlates with multiple metabolic pathways and p53 signaling pathway

**Fig. 7:**
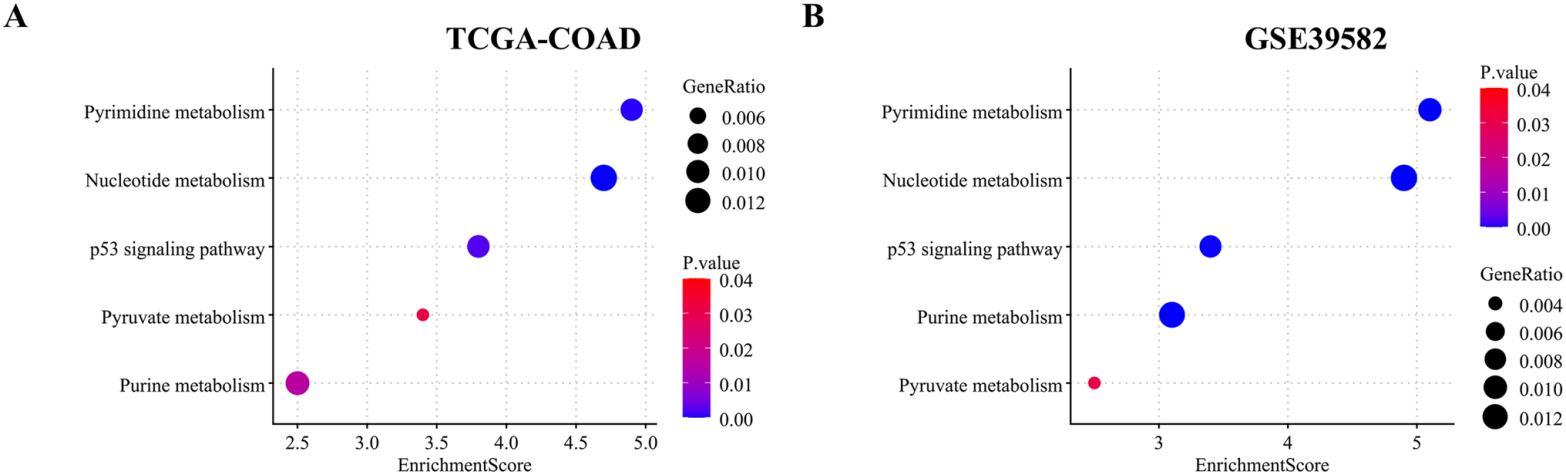
High CCT7 expression is associated with multiple metabolic pathways and p53 signaling pathway. **A** and **B** KEGG enrichment analysis of CCT7 using the TCGA-COAD **(A)** and GSE39582 **(B)** datasets

To explore the downstream signaling pathways through which CCT7 exerted its effects, KEGG enrichment analyses were performed on the TCGA-COAD and GSE39582 datasets. The results implicated CCT7 in processes including nucleotide metabolism, purine metabolism, pyrimidine metabolism, pyruvate metabolism and p53 signaling pathway (Fig. 7A and B , *P*< 0.05), suggesting potential mechanisms underlying its role in cell proliferation and cytoskeletal regulation.

## Discussion

CCT is essential for maintaining cellular protein homeostasis, as it facilitates the folding of major cytoskeletal proteins, including tubulin and actin. Research has shown that the CCT protein expression is elevated in 18 types of cancer cell lines when compared with normal paired cells (Boudiaf-Benmammar et al. 2013). Several CCT subunits have been implicated in cancer: CCT2 drives CRC proliferation and metastasis via the Hedgehog pathway under hypoxia (Park et al. 2020); HOXB2 upregulates CCT6A to enhance CRC proliferation and invasion (Yang et al. 2022a), consistent with CCT6A’s role in LUAD (Yu et al. 2024); CCT8 promotes CRC progression through the p53 pathway (Liao et al. 2021). Although bioinformatics reveals CCT7 overexpression in CRC tissues (Lim et al. 2022), its role in COAD development remains unclear. In this study, we investigated the role of CCT7 in COAD and its correlations with immunotherapy and chemotherapy responsiveness.

In line with the increased expression of CCT2, CCT5 and CCT7 in CRC tissues (Park et al. 2020; Liu et al. 2021; Ahmed et al. 2025), the present study found through analyses of the TCGA and GEO databases that the mRNA expression of CCT7 was statistically significantly elevated in COAD tissues compared with normal samples. Similar results were observed in colon cancer cells, normal colon cells, and COAD clinical specimens using qRT-PCR, western blotting, and IHC techniques. In addition, Kaplan–Meier and log-rank methods showed that high CCT7 expression was associated with worse prognosis in COAD patients and Cox regression analysis demonstrated that high CCT7 expression was an independent predictor of poor prognosis, which was consistent with the prognostic value of CCT2 in breast cancer, CCT5 in gastric cancer and CCT3 in head and neck squamous cell carcinoma (HNSCC) and cervical cancer (Dou and Zhang 2021; Wang et al. 2021; Li et al. 2022; Chen et al. 2024a). Collectively, these results suggest that CCT7 may function as an oncogene in COAD and could serve as a potential prognostic biomarker for COAD patients.

To define the detailed functions of CCT7 in COAD, GO-BP enrichment analysis was used to identify CCT7-related biological processes. The results revealed that CCT7 may play a role in regulating cell proliferation and microtubules-related activities in COAD. Microtubules, as essential tracks for transporting organelles and molecules via motor proteins, facilitate cell migration by delivering key proteins and signaling molecules to the cell front. During cell migration, microtubules and actin work together to transfer mechanical forces: microtubules provide flexural stiffness, while actin filaments generate contractile force, allowing cells to move efficiently. To further validate the aforementioned results, the effects of CCT7 on biological behaviors of COAD cells were examined.

It has been reported that CCT2, CCT3 and CCT8 promote the proliferation of tumor cells in breast cancer, HNSCC, cervical cancer and CRC respectively (Xu et al. 2020; Dou and Zhang 2021; Liao et al. 2021; Wang et al. 2021; Chen et al. 2024a). Likewise, our functional experiments showed that knockdown of CCT7 suppressed proliferation of COAD cells, which was consistent with the results of enrichment analysis. Moreover, CCT2, CCT3 and CCT8 have been shown to enhance the migration of cancer cells in breast cancer, HNSCC, cervical cancer and CRC respectively (Xu et al. 2020; Dou and Zhang 2021; Liao et al. 2021; Wang et al. 2021; Chen et al. 2024a). Similar effects of CCT7 on COAD cell migration were observed in the present study, also in agreement with enrichment analysis. In addition, our results further demonstrated that CCT7 enhanced the invasive capacity of COAD cells, a functional role analogous to those of CCT2, CCT3 and CCT8 in breast, cervical, and colorectal cancers, respectively (Dou and Zhang 2021; Liao et al. 2021; Chen et al. 2024a). Cancer cell invasion involves growth, migration, and proteolytic degradation of tissue barriers like the extracellular matrix (ECM) and basement membrane (Kapinova et al. 2018), and requires MMPs to hydrolyze and degrade the ECM and basement membranes of surrounding normal tissues (Wu et al. 2016). Studies have demonstrated that inhibiting MMP1 and MMP2 reduces CRC cell migration in vitro (Tian et al. 2025; Xu et al. 2025), and experiments have shown significantly elevated MMP9 levels in CRC tumor tissues, with MMP9 overexpression being a critical event in tumor metastasis (Veljkovic et al. 2025). Furthermore, CCT6A plays a crucial role in regulating neuronal protrusion migration dependent on MMP3 (Van Hove et al. 2015). Based on these findings, we speculate that MMPs may be involved in the stimulatory effect of CCT7 on COAD cell invasion, though further research is needed. Taken together, these results demonstrate that high CCT7 expression is associated with malignant progression of COAD. Combined with the findings regarding the prognostic significance of elevated CCT7 expression in COAD patients, the roles and mechanisms of CCT7 in the tumorigenesis and progression of COAD have been partially elucidated.

Furthermore, this study investigated the influence of CCT7 expression on the efficacy of immunotherapy and chemotherapy. Results from ssGSEA based immune infiltration analysis and correlation analysis revealed a negative correlation between CCT7 expression and the infiltration of most immune cells, as well as a negative correlation with the expression levels of most immune cell markers. These findings suggest that high expression of CCT7 may reduce immune cell infiltration, thereby compromising the effectiveness of immune-activating agents in the therapy of COAD. Additionally, CCT7 expression showed a negative correlation with PD-L1, PD-L2, CTLA-4, CD80, and CD86, and was not associated with the predicted scores for PD-1 and CTLA-4 inhibitor therapies, indicating that CCT7’s value as a predictive biomarker for ICI therapy in COAD remains unclear. These findings provide a basis for further investigation of CCT7-targeted therapy in combination with immunotherapy.

Drug sensitivity analysis demonstrated that COAD samples with high CCT7 expression exhibited increased sensitivity to 5-Fluorouracil and Oxaliplatin, both of which are well-established clinical agents for COAD treatment (Shaham et al. 2025). Consequently, high CCT7 expression may enhance the therapeutic efficacy of conventional clinical drugs for COAD. Moreover, our results also indicated that COAD samples with high CCT7 expression showed increased sensitivity to OSI-027, VX-11e, MK-1775, Palbociclib, and Vorinostat, while showing decreased sensitivity to AZD6482, AZD8186, NU7441, ZM447439, JQ1, and Doramapimod. These findings provide important insights for clinical drug selection.

The dynamics of microtubules are closely coupled with cellular metabolism and signaling pathways through multiple molecular mechanisms. Specifically, nucleotide metabolism directly regulates the polymerization and depolymerization of microtubules through the interconversion of GTP and GDP, driving microtubule instability (Weisenberg et al. 1976). Purine metabolism not only supplies GTP for microtubule assembly but also undergoes directional transport along the microtubule track through multi-enzyme complexes to optimize metabolic flux spatially (Chan et al. 2018). At the pharmacological level, pyrimidine derivatives can bind to taxol sites of microtubule proteins, stabilizing or depolymerizing microtubules as anti-cancer strategy (Sáez-Calvo et al. 2017). In pyruvate metabolism, pyruvate kinase binds directly to the C-terminal tail of tubulin and induces microtubule depolymerization, establishing a bidirectional interaction between metabolic flux and cytoskeletal reorganization (Orosz et al. 1999). Additionally, p53 can participate directly in regulating microtubule composition and function (Galmarini et al. 2003). In our study, bioinformatics analysis revealed that CCT7 is associated with multiple metabolic pathways and p53 signaling pathway. Studies have shown that in CRC, dysregulated nucleotide metabolism induces immunosuppression by impairing immune cell function, attenuating intratumoral infiltration, and activating immune checkpoint pathways, thereby facilitating immune evasion and 5-fluorouracil resistance (Zhao et al. 2024; He et al. 2025). Additionally, Wnt/β-catenin signaling transcriptionally reprograms purine metabolism to promote oxaliplatin resistance (Chen et al. 2025). Dysregulated pyrimidine metabolism further contributes to cancer cell proliferation, survival, and chemoresistance (Nadhan et al. 2023). Aberrant pyruvate metabolism sustains the TME and enables immune evasion (Shi et al. 2023). As a central tumor suppressor, p53 induces cell-cycle arrest, and inhibits proliferation (Zhan et al. 2017), and its inactivation in CRC unleashes oncogenic signaling pathways to promote tumor progression (Ma et al. 2019).

Modern biotechnology contributed extensively to cancer research. In metabolic engineering, introducing the human hypoxia-inducible factor-1 (HIF-1) complex into yeast simulates cancer-like metabolic reprogramming, such as the Warburg effect, enhancing triterpenoid biosynthesis (Lin et al. 2023). This advances cancer metabolism research and provides potential anti-cancer drug sources. In genomic analysis, high-throughput sequencing studies reveal no significant link between somatic clonal expansion in normal tissues and cancer risk across organs (Zhang et al. 2024). This indicates they might be distinct evolutionary processes, offering new insights into cancer initiation. Overall, modern biotechnology has established a multi-dimensional research framework for more accurate prediction and intervention in cancer progression.

There are several limitations to our study. Firstly, the signaling pathways responsible for the effect of CCT7 on the biological behaviors of COAD cells should be identified in future research. Secondly, the associations of CCT7 expression with immune infiltration and immunotherapy score were determined based on bioinformatic databases and therefore, in vivo and in vitro experiments should be employed to investigate the effect of CCT7 on the infiltration of immune cells, immunotherapy and the underlying mechanisms. Thirdly, in vivo and in vitro experiments should be conducted to confirm the influence of CCT7 expression on chemotherapy, as the impact of CCT7 on drug sensitivity was obtained only through bioinformatics analysis. Fourthly, although all the IHC results were evaluated by two independent pathologists using a unified scoring standard, the assessment remained a subjective judgment and deviation may exist. Furthermore, the sample size of this IHC-related study was small, and a large sample size is required to verify our findings. Fifthly, retrospective studies may miss critical data and be affected by confounding factors. Sixthly, bioinformatics analysis relies on public databases, and the data quality varies; therefore, predictions such as immune infiltration, immunotherapy score and drug sensitivity require experimental validations. Seventhly, computational immune deconvolution also has limitations, as the complexity of gene expression signals makes it difficult to fully resolve the contributions of individual cell types. Lastly, regarding tumor purity and stromal content, low-purity samples may underestimate immune cell abundance, and overlap between stromal and immune cell signals may generate false correlations.

## Conclusion

In summary, our study demonstrates that the elevated expression of CCT7 in COAD contributes to enhanced tumor proliferation, migration, and invasion, ultimately affecting patient prognosis. Furthermore, the expression level of CCT7 influences the predicted efficacy of both immunotherapy and chemotherapy, suggesting that CCT7 may serve as a useful biomarker guiding clinical management and therapeutic decision-making in COAD.

## Data Availability

The datasets used and/or analyzed during the current study are available from the corresponding author upon reasonable request.
